# Effect of the bovine *TG5* gene polymorphism on milk- and meat-producing ability

**DOI:** 10.14202/vetworld.2020.2046-2052

**Published:** 2020-10-02

**Authors:** I. Dolmatova, T. Sedykh, F. Valitov, R. Gizatullin, D. Khaziev, A. Kharlamov

**Affiliations:** 1 Federal State Budgetary Educational Institution of Higher Education, Bashkir State Agrarian University, Ufa, Russia; 2 Ufa Branch of the Russian Academy of Sciences, Bashkir Scientific Research Institute of Agriculture, Ufa, Russia; 3 Federal State Budgetary Scientific Institution, Federal Scientific Center for Biological Systems and Agrotechnologies of the Russian Academy of Sciences, Orenburg, Russia

**Keywords:** alleles, dairy and meat productivity, genetic polymorphism, Hereford breed, limousine breed, thyroglobulin gene

## Abstract

**Aim:**

This study aimed to determine the effect of thyroglobulin (*TG5*) gene polymorphism on milk and meat productivity in the various cattle breeds currently bred in the Republic of Bashkortostan.

**Materials and Methods:**

The test was performed on dairy cattle of Black-and-White, Bestuzhev, and Simmental breeds, and meat cattle of Hereford and limousine breeds. The purpose of the test was to search for associations between the polymorphic alleles of the thyroglobulin (*TG5*) gene and economically useful traits.

**Results:**

All studied breeds showed a frequency predominance of the *TG5^C^* allele (from 0.56 to 0.71). A clear trend of an effect of the genotypes of the *TG5* gene on milk-productivity indicators was revealed; cows with the *TG5^TT^* genotype have the highest milk yield and fat content in milk. The milk of cows of Bestuzhev and Simmental breeds that possessed this genotype was also characterized by higher protein content.

**Conclusion:**

We identified an effect of the polymorphism of the *TG5* gene in the Hereford and limousine breeds on fat metabolism intensity indicators, such as fat output and fat content, in the longissimus muscle and in the general sample of ground beef.

## Introduction

At present, domestic and foreign scientists are actively studying the genetic determination of the dairy and meat production characters of cattle using DNA markers. This will allow supplementing traditional breeding methods with the method of selecting animals based on desirable genetic markers. Thus, it will become possible to significantly accelerate the selection process by improving the genetic potential of cattle breeds [1,2]. It will be possible to maintain the desired economic and useful characteristics during cattle breeding by determining the quantity and quality of the products [2-4].

Polymorphic genes encoding hormones such as somatotropin, prolactin, and thyroglobulin are primarily considered candidate genes that determine the level of dairy and meat productivity of cows. The pituitary protein hormones prolactin and somatotropin are directly involved in the initiation and maintenance of lactation in mammals. The glycoprotein thyroglobulin is a precursor of the thyroid iodothyronine hormones, which modulate many physiological and biochemical processes in almost all tissues of the body by regulating gene expression. Thyroid hormones also affect the differentiation of adipocytes into adipose tissue. Therefore, the thyroglobulin gene is considered a candidate gene that affects the ability to accumulate fat in tissues, including milk [5].

The thyroglobulin 5′ leader sequence (*TG5*) gene is one of the longest mammalian genes. In cattle, it is located in the centromere region of the 14^th^ chromosome and consists of 37 exons [6-9]. The *TG5* gene has two allelic variants, *TG5^T^* and *TG5^C^*, and three genotypes, *TG5^CC^*, *TG5^CT^*, and *TG5^TT^*.

The analysis of the research results of domestic and foreign colleagues confirmed that the *TG5* gene should be considered a functional and positional candidate gene that affects the accumulation of fat in the body. In fact, a single-nucleotide polymorphism located in the 5′ untranslated region of this gene is used for marker-based selection aimed at increasing marbling [10]. Several studies have revealed an association between thyroglobulin gene polymorphism and fat metabolism, particularly intramuscular fat [11-15], as well as milk yield and quality [11,16,17]. Thus, cows of the Kholmogorsky breed of the Tatarstan type carrying the *TG5^CT^* and *TG5^TT^* genotypes surpassed their age equals carrying the *TG5^CC^* genotype regarding milk yield and mass fraction of fat in milk. On average, more milk fat per lactation was obtained from the first-born cows of different breeds with the *TG5^CT^* and *TG5^TT^* genotypes compared with cows with the *TG5^CC^* genotype.

Despite the identification of several trends in this field of research, it is evident that the introduction of marker-based selection in practical cattle breeding requires an improved analysis of the gene pool of domestic breeds. This will help increase the intensity of the improvement of the genetic potential of domestic breeds of cattle.

This study aimed to determine the effect of *TG5* gene polymorphism on milk and meat productivity in the various cattle breeds currently bred in the Republic of Bashkortostan.

## Materials and Methods

### Ethical approval

Housing conditions for the animals in the experimental groups were the same. Investigational study and animal management meet the requirements of the Russian Regulations, 1987 (Order No. 755 on 12.08.1977 the USSR Ministry of Health) and «The Guide for Care and Use of Laboratory Animals (National Academy Press Washington, D.C. 1966)››. Every possible effort was made during the research to minimize animals’ distress and use fewer samples.

### Study period

The subject of the research made from 2014 to 2018 was milk and meat-type cattle population of the Republic of Bashkortostan.

### Animals and location of the study

First-calf cows of the Black-and-White (Limited Liability Company [LLC] Agricultural Enterprise named after the Kalinina Sterlitamakskii district; n=379), Bestuzhev (LLC Agrofirma “Idel,” Nurimanovskii district; n=150), and Simmental (LLC JV “Trudovik,” Meleuzovskii district; n=150) breeds were used as the material for on the study of the relationship between the *TG5* gene polymorphism and milk productivity.

The samples were formed using the method of balanced groups, considering the date of birth and the calving date (first lactation). Milk productivity data were obtained from the electronic database of the “Selex” enterprise information system. The analysis of milk composition was performed in the milk quality selection control laboratory of “Bashkirskoe” JSC, which specializes in breeding programs based on the use of a “Lactan 700M” device. The amount of milk fat was calculated by dividing one percent milk obtained over the considered lactation period by 100.

The analysis of meat productivity indicators in relation to the *TG5* gene polymorphism was performed on animals of the Hereford (SAVA-Argo-Usen LLC, Tuymazinskii district; n=115) and limousine (SAVA-Agro-Iapryk LLC, Tuymazinskii district; n=114) breeds. Hereford bulls were the offspring of animals imported from Australia in 2009. Limousine bulls were the offspring of animals bred by accumulation crossing of Simmental cattle with limousine servicing bulls of French selection. Both farms use stable and open-grazing keeping of meat cattle according to the “cow−calf” system with resource-saving elements. The farms were stud farms that specialize in particular breeds of unique meat cattle and maintain feeder animals for beef production. Bulls were raised and fed until the age of 20 months. Live-animal indicators of meat productivity (bodyweight of young animals at birth, absolute and average daily weight gain, and relative growth rate over the entire growing period) and gained bodyweight (bodyweight at the end of fattening of young animals), as well as postmortem indicators, were considered in the determination of meat quality during the experiment.

### Sample collection and analysis

DNA samples were obtained from whole blood using a set of “DNA-Extran” (“Syntol” LLC). Genotyping of the *TG5* gene was performed in the animals through the PCR−RFLP method using the following primers [18]:

*TG5 1*: 5′−GGGGATGACTACGAGTATGACTG−3′
*TG5 2*: 5′−GTGAAAATCTTGTGGAGGCTGTA−3′

The resulting amplified fragments of the *TG5* gene were cleaved by the BstX2I endonuclease at 37°C for 12 h. Electrophoresis of restriction products was performed in 7% polyacrylamide gels with the addition of ethidium bromide in 0.5× TBE buffer. The Doc XR gel documentation system and the Image Lab software, 2.0 “DNA-analyzer” version, were used to analyze the gel images. The molecular mass marker used was the 100 bp + 1 kb marker provided by the Sibenzim company.

The formula determined the genotype frequency:

*p*=*n*⁄*N* (1)

where *p* is the genotype frequency;

*n* – the number of animals with a specific genotype;

*N* – number of animals.

The frequency of individual alleles was determined using the following formulas:

*RS*=(2*pss*+*PST*)⁄2*N* (2)

where *pC* is the frequency of *the C* allele.

*qT*=(2*ptt*+*PST*)⁄2*N* (3)

where *qT* is the frequency of the *T* allele.

The expected degree of heterozygosity was calculated (H_e_) using the formula:


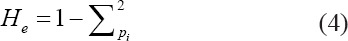


where *p_i_* is the i-th allele frequency.

The observed level of heterozygosity (*H_o_*) was calculated using the formula:

*H_o_*=*n⁄N* (5)

where *n* is the number of animals that are heterozygous for this allele,

*N* is the sampling size.

For assessing the correspondence of the actual and expected distribution of genotypes in the studied animal samples, the χ*2* criterion was used, which was calculated using the formula:


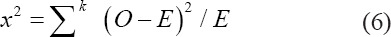


where *O* and *E* are the observed and theoretically expected numbers of genotypes of a particular type,

*k* is the number of genotypic classes.

Statistical processing of the quantitative indicators of dairy and meat productivity in animals was performed using a standard method and the “Excel” software of the “Microsoft Office” package.

## Results and Discussion

**Figure-1:**
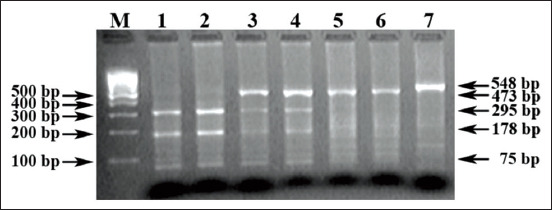
Electrophoregram of the result of TG5 gene identification of cattle.

Figure-1 shows an electropherogram of the results of PCR−RLFP of the *TG5* gene. The *TG5^CC^* genotype was represented by restriction fragments of 295, 178, and 75 bp (tracks 1 and 2); the *TG5^TC^* genotype was represented by 473, 295, 178, and 75 bp fragments (tracks 3 and 4); and the *TG5*^TT^ genotype was represented by 473 and 75 bp fragments (tracks 5, 6, and 7).

The frequency of the *TG5* genotypes in the animals of the studied breeds is shown in Table-1. It should be noted that, in general, the *TG5* genotypes were distributed similarly among the four studied breeds, with the exception of Simmental. Specifically, the frequency of the *TG5^CC^* genotype was very high, ranging from 47.32% to 65.8%. The heterozygous *TG5^CT^* genotype ranked second in frequency (27.9-43.0%), and the *TG5^TT^* genotype ranked third (6.3-13.4%) in frequency. In contrast, more than half of the studied animals of the Simmental breed (62.7%) had the *TG5^CT^* genotype, whereas 23.2% carried the *TG5^CC^* and 14.1% had the *TG5^TT^* genotypes.

**Table 1:** Genotype frequencies for the *TG5* gene.

Breed	*n*	The frequency of the genotype					

		*TG5^CC^*		*TG5^CT^*		*TG5^TT^*	
		
		Animals	%	Animals	%	Animals	%
Black-and-White	379	186	49.0	163	43.0	30	8.0
Bestuzhev	150	99	65.8	42	27.9	9	6.3
Simmental	150	35	23.2	94	62.7	21	14.1
Hereford	115	60	52.17	44	38.26	11	9.57
Limousine	112	53	47.32	44	39.28	15	13.40

**Figure-2:**
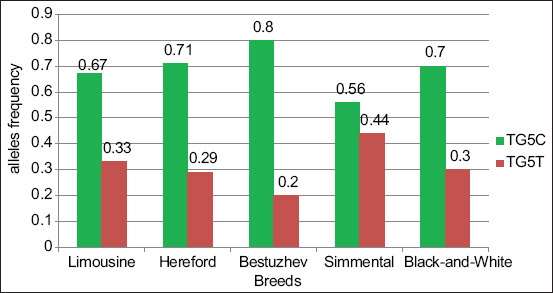
TG5 gene allele’s frequency.

The *TG5* gene allele frequencies in the various breeds of cattle are shown in Figure-2. The analysis of the graphic data revealed that the frequency of the *TG5^C^* allele was high in all breeds, especially in the Bestuzhev (0.80), Hereford (0.71), and Black-and-White (0.70) breeds.

The data obtained were in good agreement with the results of domestic research. Soloshenko *et al*. [19] found that, among the Hereford cattle bred in Siberia, 65%−76% of animals have the *TG5^CC^* genotype, 20-35% carry the *TG5^TC^* genotype, and 2% have the *TG5^TT^* genotype (identified only in one herd). The study of the Hereford cattle bred in Tatarstan [20] showed that none of the animals carried the *TG5^TT^* genotype. In limousine cattle, the frequency of the *TG5^TT^* genotype was 22.6%, whereas the *TG5^CT^* genotype was present in 29% of these animals and in 11.1% of Hereford cattle. Moreover, a population-genetics analysis of beef cattle bred in Ukraine revealed that the frequency of the *TG5^CT^* genotype in the Hereford breed was 0.23% [21]. According to Barends*e et al*. [7], the highest marbling was noted in the Japanese Wagyu breed, in which the frequency of the desired TT genotype reaches 76%.

According to Tiul’kin *et al*. [16], 62.9% of purebred and crossbred Holstein bulls have the *TG5^CC^*, 35.7% possess the *TG5^CT^*, and only 1.4% have the *TG5^TT^* genotype. No animals with the *TG5^TT^* genotype were found in a herd of Black-and-White Holstein cows.

Thus, the analysis of the frequency of the various genotypes of the *TG5* gene indicates the presence of genetic potential among specialized breeds of beef cattle in relation to the taste and nutritional qualities of meat. The foreign origin of beef cattle can explain the relatively high proportion of animals with the desired *TG5^TT^* genotype identified here.

The actual and expected levels of heterozygosity of the *TG5* gene among the studied samples are shown in Table-2. Out of the three dairy breeds, only the Black-and-White breed was in the state of genetic equilibrium for the *TG5* gene. The actual number of heterozygotes in this breed corresponded to the theoretically expected number calculated according to the Hardy−Weinberg law. The most significant deviation from the actual and expected distribution of genotypes was found in the Simmental breed sample, which exhibited a relatively significant excess of *TG5^CT^* heterozygotes. This caused some lack of both *TG5^CC^* and *TG5^TT^* homozygous genotypes. In the Bestuzhev breed, in contrast, a borderline (close to the standard table value of −5.99) value of the χ^2^ (6.09) criterion was found because of the slight lack of heterozygotes.

**Table 2:** Actual and expected levels of heterozygousness by the *TG5* gene.

Breed	H_o_	H_e_	F	χ^2^
Black-and-White	0.430	0.420	0.010	0.575
Bestuzhev	0.279	0.320	–0.041	6.09
Simmental	0.627	0.493	0.134	10.66
Hereford	0.382	0.412	–0.030	0.218
Limousine	0.393	0.442	–0.049	0.543

H_o_ is the observed heterozygousness; H_e_ is the expected heterozygousness; F-is the difference of H_o_-H_e_; + or - is the excess/deficit of heterozygotes; χ^2^ is the criterion of matching of the observed and expected distribution of genotypes

In the Hereford and Limousine breeds, the indicators of expected heterozygosity for the *TG5* gene differed slightly. The Pearson criterion values were not large, which indicates that the population was in a state of genetic balance.

The results of the analyses of the milk productivity and technological properties of the milk of Black-and-White cows of Bestuzhev and Simmental breeds with different genotypes of the *TG5* gene are presented in Table-3. Animals of all three breeds carrying the *TG5^TT^* genotype exhibited the best indicators of milk yield and milk fat. Thus, among the Black-and-White cows of this genotype, the highest milk yield was 4825.3 kg and the highest fat content of milk was 3.78 %. The difference in milk yield between the *TG5^CC^* and *TG5^TT^* genotypes was 254.6 kg. This difference was significant.

**Table 3:** Qualitative indicators of milk from cows with different genotypes for the *TG5* gene.

Indicator	Genotype			Difference between genotypes		
	
	*TG5^CC^*	*TG5^CT^*	*TG5^TT^*	*TG5^CC^*/*TG5^CT^*	*TG5^CC^*/*TG5^TT^*	*TG5^CT^*/*TG5^TT^*
Black-and-white breed (n=50)						
Milk yield, kg	4570.7±89.0	4756.4±99.7	4825.3±95.9	–185.7	–254.6*	–66.9
Fat, %	3.69±0.05	3.75±0.02	3.78±0.04	–0.06	–0.09	–0.03
Protein, %	3.28±0.07	3.26±0.01	3.25±0.08	0.02	0.03	0.01
Casein, %	2.77±0.10	2.88±0.05	2.83±0.09	–0.11	–0.06	0.05
Lactose, %	4.95±0.15	4.99±0.04	4.97±0.11	–0.04	–0.02	0.02
MSNF, %	8.91±0.17	8.97±0.22	8.95±0.18	–0.06	–0.04	0.02
Milk fat, kg	168.6±5.2	178.3±4.5	182.4±4.9	–9.7	–13.8*	–4.1
Bestuzhev breed (n=50)						
Milk yield, kg	3428.4±88.5	3450.1±78.9	3581.2±56.9	–21.7	–152.8	–131.1
Fat, %	3.50±0.07	3.57±0.09	3.88±0.06	–0.07	–0.38**	–0.31**
Protein, %	3.22±0.06	3.26±0.07	3.47±0.03	–0.04	–0.25**	–0.21**
Casein, %	2.80±0.17	2.76±0.11	2.85±0.10	0.04	–0.05	–0.09
Lactose, %	4.64±0.12	4.25±0.14	4.86±0.10	0.39	–0.22	–0.61**
MSNF, %	8.56±0.15	8.66±0.17	9.03±0.26	0.1	–0.47	–0.37
Milk fat, kg	119. 9±6.1	123.2±4.9	138.9±5.1	–3.3	–19.0*	–15.7*
Simmental breed (n=50)						
Milk yield, kg	3785.5±88.5	3829.6±80.6	3975.2±56.9	–24.1	–189.7*	–145.6
Fat, %	3.66±0.08	3.62±0.09	3.92±0.09	0.04	–0.26*	–0.3*
Protein, %	3.37±0.07	3.29±0.08	3.56±0.06	0.08	–0.19*	–0.27*
Casein, %	2.90±0.19	2.86±0.12	2.95±0.11	0.04	–0.05	–0.09
Lactose, %	4.52±0.13	4.50±0.16	4.55±0.14	0.02	0.03	0.05
MSNF, %	8.74±0.17	8.80±0.23	9.03±0.17	–0.06	–0.29	–0.23
Milk fat, kg	138.5±7.0	138.6±6.5	155.8±4.8	–0.1	–17.3*	–17.2*

MSNF=Milk solids non-fat, Significance of differences according to Student’s t test

* -p<0,05;

** -p<0,01

No consistent differences in the mass fraction of fat were found in the milk of cows of different genotypes. However, this indicator was higher among animals with the *TG5^TT^* genotype. Black-and-White cows with this genotype had a higher yield of milk fat (13.8 kg higher than that of cows with the *TG5^CC^* genotype). No other consistent differences in milk quality indicators were observed.

Among cows of the Bestuzhev and Simmental breeds, the tendency of the *TG5* genotype to affect the milk productivity indicators remains. Thus, cows of both breeds with the *TG5^TT^* genotype have the highest milk yield and milk fat content (in Bestuzhev and Simmental cows, the difference in relation to the *TG5^CC^* genotype was 152.8 and 189.7 kg, respectively, and the mass fraction of fat in milk was 0.38% and 0.26%, respectively). The fact that the *TG5^TT^*genotype was associated with higher protein content in the milk of Bestuzhev and Simmental breed cows was unexpected. The difference in favor of this genotype compared with the *TG5^CC^* and *TG5^CT^* genotypes was 0.25% and 0.21% in the Bestuzhev breed cows, and 0.19%, 0.27% in the Simmental breed cows, respectively. In terms of milk fat output, cows with the *TG5^TT^* genotype also exhibited a favorable difference.

The data obtained here were in accordance with the results of Kharzinova *et al*. [22]. According to Kharzinova *et al*. [22], Black-and-White cows with the *TG5^CT^* genotype have the best milk productivity indicators. Concomitantly, Zinnatova and Zinnatov [17] studied the same breed and found no significant differences between genotypes related to milk productivity indicators.

Table-4 shows the live and postmortem indicators of meat productivity of Hereford and limousine animals with different genotypes of the *TG5* gene. The analysis of the indicators presented in Table-4 led us to conclude that calves with different genotypes had the same birth weight. However, at the end of fattening, the bodyweight of bulls with the *TG5^CC^* genotype was increased compared with the bulls with *TG5^CT^* and *TG5^TT^* genotypes. Among Hereford breed bulls with these genotypes, the removable body weight was higher by 1.16% and 2.04%, and for the Limousine breed, it was higher by 6.65% and 9.53%, respectively. The animals with the *TG5^CC^* genotype studied here also had higher values of absolute and average daily gains in body weight, pre-slaughter body weight, and carcass yield. These indicators increased in the following order of genotype: *TG5^CC^* > *TG5^CT^* *>* *TG5^TT^*.

**Table 4:** Meat productivity and quality of meat of bulls of various genotypes by the *TG5* gene.

Indicator	Genotype		

	*TG5^CC^* (n=20)	*TG5^CT^* (n=20)	*TG5^TT^* (n=10)
Hereford breed (n=50)			
Bodyweight of newborn calves, kg	33.5±0.28	33.1±0.35	33.0±0.42
Gained bodyweight, kg	576.25±5.74	569.6±5.42	564.7±6.18
Absolute body gain, kg	542.8±5.22	536.5±6.36	531.7±6.33
Average daily gain of bodyweight, g	892.8±8.59	882.4±10.46	874.5±10.97
Before slaughter bodyweight, kg	559.9±5.80	552.4±3.06	549.4±5.20
Carcass yield, %	58.8±0.53	58.7±0.42	58.4±0.60
Internal raw fat weight, kg	19.09±0.51	19.15±0.32	20.09±0.30
Fat yield, %	3.40±0.09	3.40±0.05	3.65±0.07*
Slaughter yield, %	62.20±0.24	62.10±0.27	62.00±0.18
The fat content of the rib eye,%	5.70±0.21	6.05±0.90	6.44±0.15*
Fat content in the general sample of ground beef, %	14.38±0.10	14.54±0.08	14.69±0.06*
Limousine breed (n=50)			
Bodyweight of newborn calves, kg	33.9±0.30	34.2±0.27	34.9±0.23
Gained bodyweight, kg	604.1±3.99	598.1±2.16	581.4±6.60
Absolute bodyweight gain, kg	570.2±4.89	563.9±3.07	546.5±4.47
Average daily bodyweight gain, g	937.8±8.05	927.5±5.05	898.9±7.34
Before slaughter bodyweight, kg	584.0±6.16	577.4±0.44	567.0±4.43
Carcass yield, %	60.0±0.62	59.9±0.96	59.9±0.78
Internal raw fat weight, kg	17.30±0.18	17.80±0.17	18.00±0.33
Fat yield, %	2.95±0.01	3.06±0.14	3.19±0.02*
Slaughter yield, %	62.90±0.11	63.10±0.12	63.10±0.22
The fat content of the rib eye,%	5.60±0.28	5.95±0.28	6.55±0.16*
Fat content in the general sample of ground beef, %	13.16±0.05	13.46±0.03	13.65±0.26*

Significance of differences according to Student’s t test

* -p<0,05

Bulls with the *TG5^TT^* genotype for both of the studied breeds had higher indicators of internal raw fat, fat yield, rib-eye fat content, and the ground beef sample compared with bulls with the *TG5^CC^* genotype.

The same indicators were higher in the Hereford breed cattle by 4.98%; 0.25% (p<0.05); 0.74% (p<0.05); and 0.31% (p<0.05), respectively; in the Limousine breed, they were higher by 3.89%; 0.24% (p<0.05); 0.95% (p<0.05); and 0.49% (p<0.05), respectively. It should be noted that the Hereford cattle tended to accumulate more fat tissue, which was attributed to the biological characteristics of this breed. Carvalho *et al*. [10] performed similar studies but did not find a reliable relationship between *TG5* gene polymorphism and meat productivity indicators. However, in contrast with our results, their studies proved that the preslaughter weight in animals with the *TG5^TT^* genotype tended to be increased. Our findings confirmed a higher fat yield in animals with the *TG5^TT^* genotype and were consistent with the results of the study of Casas *et al*. [23]. The increase in fat content in the rib eye is also confirmed by the research of Anton *et al*. [24], Barendse *et al*. [7], and Casas *et al*. [23].

## Conclusion

The *TG5* gene polymorphism is breed-specific, as proven by the distribution of the frequency of its genotypes and alleles in cattle of the Black-and-White, Simmental, Bestuzhev, Hereford, and Limousine breeds. Dairy cattle showed a clear tendency of an effect of the *TG5* genotype on milk productivity indicators. Thus, cows with the *TG5^TT^* genotype had the highest milk yield and fat content in milk. An association between *TG5* gene polymorphism and indicators of the intensity of fat metabolism, in particular fat output and fat content in the rib eye and the total sample of ground beef, was found in cattle of both breeds.

When carrying out selection and breeding activities, it is advisable to consider *TG5* genotyping as an additional criterion for animal selection, to improve both dairy and meat characteristics.

## Authors’ Contributions

ID, TS, FV, RG, DK, AK contributed equally to the experimentation. ID, TS, and DK wrote and edited the article. FV, RG, and AK equally designed and conducted the experiment. ID, DG, and TD studied scientific literature about the topic. All authors read and approved the final manuscript.
